# Rheological Basics for Modeling of Extrusion Process of Wood Polymer Composites

**DOI:** 10.3390/polym13040622

**Published:** 2021-02-19

**Authors:** Krzysztof Wilczyński, Kamila Buziak, Adrian Lewandowski, Andrzej Nastaj, Krzysztof J. Wilczyński

**Affiliations:** 1 Polymer Processing Department, Faculty of Production Engineering, Warsaw University of Technology, 85 Narbutta, 02-524 Warsaw, Poland; kamila.buziak@pw.edu.pl (K.B.); adrian.lewandowski@pw.edu.pl (A.L.); andrzej.nastaj@pw.edu.pl (A.N.); 2 Politech Ltd., 86-031 Osielsko, Poland; wilczynski_k@wp.pl

**Keywords:** wood polymer composites, screw extrusion, modeling

## Abstract

Wood polymer composites are materials with pseudoplastic and viscoelastic properties. They have yield stress and exhibit slip during flow. Studies on extrusion and rheology, as well as on process modeling of these highly filled materials are limited. Extensive rheological and extrusion modeling studies on the wood polymer composite based on the polypropylene matrix were performed. Viscous and slip flow properties were determined (with Rabinowitsch, Bagley, and Mooney corrections) at broad (extrusion) range of shear rate and temperature, using a high-pressure capillary rheometer. Rheological models of Klein and power-law were used for flow modeling, and Navier model was applied for slip modeling. A novel global computer model of WPC extrusion with slip effects has been developed, and process simulations were performed to compute the extrusion parameters (throughput, power consumption, pressure, temperature, etc.), and to study the effect of the material rheological characteristics on the process flow. Simulations were validated experimentally, and were discussed with respect to both rheological and process modeling aspects. It was concluded that the location of the operating point of extrusion process, which defines the thermo-mechanical process conditions, is fundamentally dependent on the rheological materials characteristics, including slip effects.

## 1. Introduction

Wood polymer composites consist of thermoplastic matrix and wooden fillers, like fiber or flour. These composites are durable, stiff, and strong, and relatively cheap compared to other competitive materials. They can be applied instead of wood since they are more resistant to decay. The wood polymer composites are mainly based on the high-density polyethylene, polypropylene, and polyvinyl chloride.

The wood polymer composites market has grown significantly in the last years due to the increasing building and automotive market. Worldwide production has increased from about 2.5 million tonnes in 2012 to about 4 million tonnes in 2015. European production has reached 350,000 tonnes in 2015 [1]. In 2019, the global wood plastic composite market size was estimated at USD 5.3 billion and is expected to register a growth rate of 11.4% over the forecast period 2000–2017 [2].

The basic technology of processing of wood polymer composites is extrusion for profile production. Processing of these highly filled composites differs substantially from polymer processing which results from different thermo-rheological material characteristics, structure, etc. Limited studies were performed on extrusion and rheology of wood polymer composites. These issues were recently discussed by the authors [3,4]. A fundamental research in this area was carried out by Xiao and Tzoganakis [5,6,7,8], Li and Wolcott [9,10,11], Błędzki et al. [12], Mohanty et al. [13], Klyosov [14], Oksman Niska [15], as well as by Vlachopoulos [16,17,18], and Zolfaghari [19].

Wood polymer composites have pseudoplastic properties, and these are also viscoelastics. The viscosity decreases when shear rate and temperature are increased, and when filler content increases, the viscosity is higher. They have yield stress and exhibit slip at the walls in extrusion [5,9,16]. The slip velocity depends on the shear rate and the filler content. When the shear rate increases, the slip velocity increases, which may lead to the plug flow [17]. Increasing of filler content also promotes the plug flow [19]. Recently, extensive FEM simulation studies on extrusion with slip effects and extrusion of viscoplastics have been performed by Lewandowski and Wilczyński [20,21].

The design of the extrusion process of wood polymer composites requires a knowledge of the flow mechanism of these highly filled materials. Only a few melting observations have been reported for wood polymer composites, however. Xiao and Tzoganakis [5,6,8] intensively investigated an extrusion of a high density polyethyelene (HDPE) composite. They observed that partially filled channels were seen until the melting process was completed. However, this has not been explained by them. This phenomenon may probably result from the screw/die interactions and insufficient pressure generation in the extruder, which leads to a starvation in the screw channel. The authors also observed a slight segregation of the components that made up the HDPE/WPC blend [8]. Close to the trailing flight of the screw, mostly HDPE was seen whereas the WPC was mostly against pushing flight. Experimental data were compared to simulations of the process using a commercially available software program (unknown). It was found that predictions of the pressure profiles generated in the extruder, in general, did not agree well with those measured.

Wood polymer composites exhibit characteristic surface tearing upon exiting from an extruder die even at low shear rates, which was reported by Hristov et al. [16,17] and Santi et al. [18]. In industrial practice, die cooling is used to produce smooth profiles, and surface tearing may be also eliminated at higher shear rates and high loading of wood flour. Recently, Santi et al. [18] modeled the flow of WPC through the die trying to explain these phenomena.

**Figure 1:**
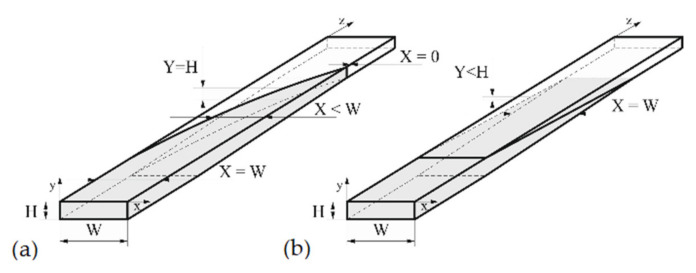
Melting mechanism: (**a**) Two-dimensional model (Tadmor model), (**b**) one-dimensional model: W—screw channel width, X—solid bed width, H—screw channel height, Y—solid bed height.

Recently, Wilczynski et al. [3] have performed extensive experimental studies on single screw extrusion of a polypropylene (PP) composite. Based on these studies, the authors developed the only available global model of extrusion of wood polymer composites [4]. According to these studies, the composite solid conveying and melting were strongly dependent on the wood filler content. The well-known classical mechanism of polymer melting developed by Tadmor et al., e.g., [22], was absent in the case of composites with high filler content (over 50%). The characteristic polymer melt “pool” was not observed at a pushing flight of the screw. However, in the case of composites with low filler content (less than 50%), this mechanism was seen. According to these observations, two models of melting have been proposed, a one-dimensional model for lower content of wood filler, and a two-dimensional model for higher content, which are schematically depicted in Figure 1 and described in [3,4]. In a classical two-dimensional model, when melting progresses, the volume of the solid material decreases by decreasing the solid width, i.e., X < W, Y = H (Figure 1a), while in one-dimensional model, the volume of the solid decreases by decreasing the solid height Y < H, X = W (Figure 1b).

When modeling extrusion process, the no-slip condition in the extruders and dies is assumed, which means the flowing polymer adheres to the wall, e.g., by White [23], Tadmor [24], or Rauwendaal [25], although this is not always obvious.

Several extrusion models have been developed using this assumption, e.g., by Agur [26], Vincelette [27], Potente [28], Wilczyński [29], and recently, e.g., [30]. The review papers on modeling of the extrusion process were delivered by Ilinca [31], Altinkaynak [32], and Malik [33]. The review contributions on global modeling of the extrusion process were delivered by Teixeira [34], Wilczyński [35,36], and Hyvärinen [37].

So far, any global model of extrusion of wood polymer composites that would include slip effects is not known. In this paper, slip effects are considered and discussed both for WPC rheological studies as well as for WPC extrusion modeling. To produce global models of the extrusion process which would include slip effects, the models for both the screw and the die need to be developed, which was discussed by Malik [33], Potente [38,39,40,41], Gooneie [42], and Duretek [43].

## 2. Material and Rheological Studies

The wood polymer composite (PP copo inj 4, Beologic) composed of the polypropylene (PP) matrix and 50% wood filler was applied in this research. The polypropylene density was equal to ρ = 0.9 g/cm^3^, the polymer melt flow index was equal to MFI = 25 g/10 min (at M = 2.16 kg, and T = 230 °C), and the melting temperature was equal to T_m_ = 160 °C. The composite melt density was equal to ρ_m_ = 0.95 g/cm^3^, the composite bulk density ρ_b_ = 0.4–0.6 g/cm^3^, and the polymer melt flow index was equal to MFI = 4 g/10 min (at M = 5 kg, and T = 190 °C).

**Figure 2:**
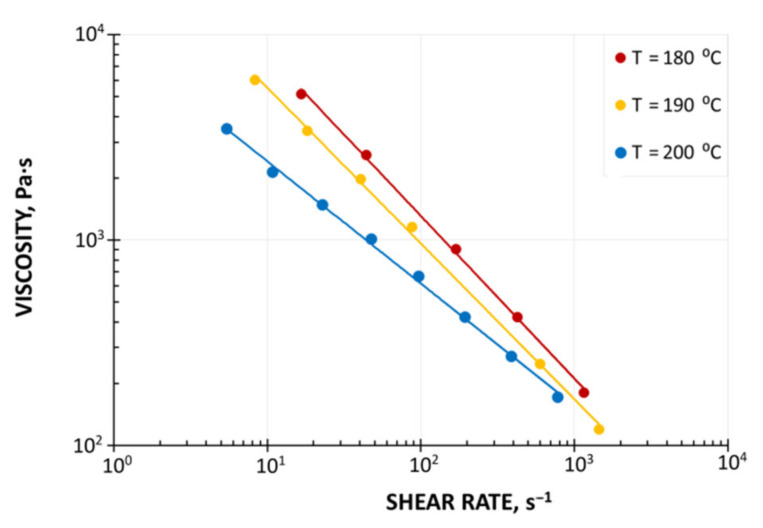
Material characteristics (PP copo inj 4, Beologic): Viscosity curves.

**Figure 3:**
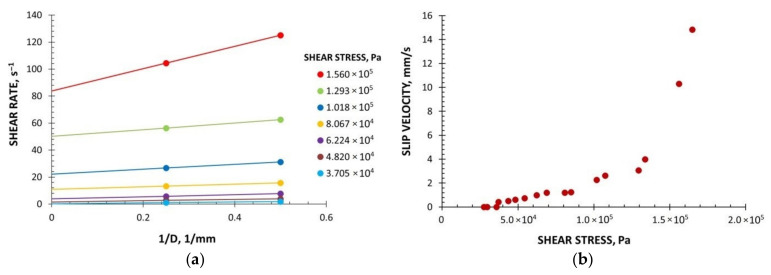
Material characteristics (PP copo inj 4, Beologic), Mooney analysis: (**a**) Shear rate vs. 1/D, (**b**) slip velocity vs. shear stress, D—capillary diameter.

**Figure 4:**
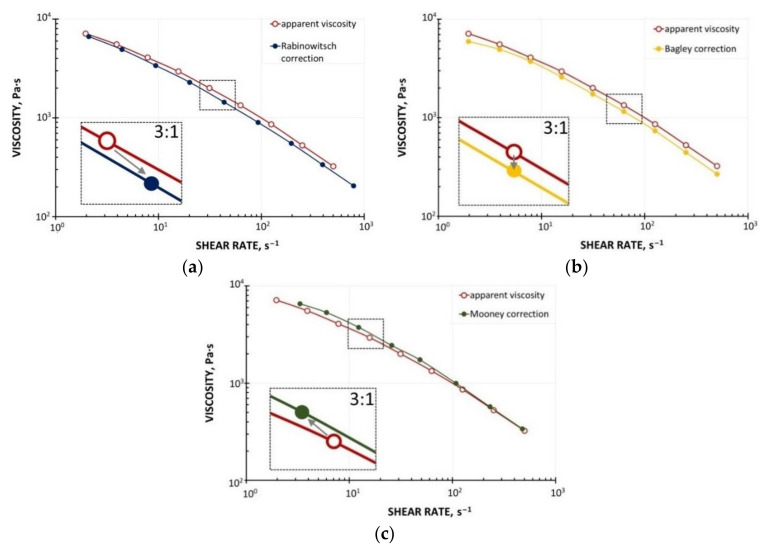
Effect of rheometric corrections on viscosity curves (PP copo inj 4, Beologic): (**a**) Rabinowitsch correction, (**b**) Bagley correction, (**c**) Mooney correction.

The WPC viscous properties have been measured at various temperatures with the use of a capillary rheometer (RG-25, Goettfert, Buchen, Germany). The capillaries of diameter D = 1, 2, 4 mm, and the length/diameter ratio L/D = 0/1, 0/2, 0/4, 10/1, 20/2, and 40/4 were used, and the Rabinowitsch correction, the Bagley correction, and the Mooney correction have been applied [44]. The measurements have been carried out at the temperatures T = 180 °C, 190 °C, and 200 °C, and at the shear rates from $\dot{\gamma }$ = 1 s^−1^ to $\dot{\gamma }$ = 1000 s^−1^. The examples of viscosity characteristics at the temperatures T = 180 °C, 190 °C, 200 °C are shown in Figure 2. An obvious pseudoplastic behavior is seen, which means the viscosity decreases with increasing the shear rate. The viscosity also decreases with increasing the temperature. However, the effect of temperature on the viscosity is not significant. The results of Mooney analysis are depicted in Figure 3, where the shear rate vs. 1/D (Figure 3a) and the slip velocity vs. shear stress (Figure 3b) are shown. The effects of the corrections of Rabinowitsch, Bagley, and Mooney on the viscosity curve are presented in Figure 4. Rabinowitsch correction means an increase of the shear rate at the constant shear stress (the constant capillary pressure drop), which results in shifting the viscosity curve shown in Figure 4a. Bagley correction means a decrease of the shear stress (the pressure decrease due to the pressure inlet loss) at the constant shear rate, which results in a shift in the viscosity curve depicted in Figure 4b. Mooney correction means a decrease of the shear rate at the constant shear stress (the constant capillary pressure drop), which results in shifting the viscosity curve shown in Figure 4c.

Two characteristic regions of slip behavior are seen in Figure 3. At the low shear stress, the slip can be characterized as weak, which is followed by a sharp increase in the slip velocity at the high shear stress, which was also observed, e.g., by Hristov et al. [16].

It is worth noting that the viscosity of composites may be evaluated based on the viscosity of the matrix and the filler content using the well-known equations of Einstein/Batchelor or Krieger/Dougherty, as it was done by Le Moigne et al. [45] and Polychronopoulos et al. [46].

## 3. FEM Flow Modeling

Studies on extrusion modeling with slip effects were mainly based on the works of Hatzikiriakos and Dealy [47], and Hatzikiriakos et al. [48,49,50].

For the slip analysis, a power-law model is usually applied which approximates the actual slip behavior of various fluids, including molten polymers [50].

(1) $$u_{s}=\beta \tau_{w}^{b}$$

where *u_s_* is the slip velocity, *β* is the slip coefficient, *b* is the slip power-law exponent, *τ_w_* is the shear stress.

In this paper, the CFD software Polyflow [51] was used for modeling to develop screw pumping characteristics which may be implemented into the global model of the extrusion process.

The modeling procedures to build the correct geometrical model of the flow, to select the proper boundary conditions and evolution schemes, as well as the meshing concepts and procedures, were discussed in [20].

**Figure 5:**
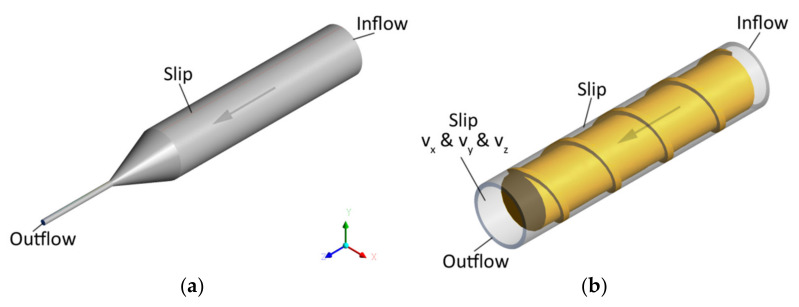
The boundary conditions for flow simulations: (**a**) Die flow, (**b**) screw flow.

The scheme of modeling and boundary conditions are depicted in Figure 5, where;

BC1—Inflow (Q_in_) = (Q_0_), the flow rate Q_0_ is imposed at the inlet of the domain,

BC2—Outflow, which means that vanishing normal forces and tangential velocities are imposed at the outlet (f_n_ & v_s_) = (0, 0),

BC3—Slip at the die/barrel wall, i.e., vanishing normal velocity is imposed, while the tangential force is a function of (v_s_ − v_wall_), i.e., f_s_ = f (v_smn_ − v_wall_), (v_n_ & f_s_) = (0, f (v_s_ − v_wall_) & v_wall_ = 0),

BC4—Slip at the screw, i.e., vanishing normal velocity is imposed, while the tangential force is a function of (v_s_ − v_wall_), i.e., f_s_ = f (v_s_ − v_wall_), (v_n_ & f_s_) = (0, f (v_s_ − v_wall_) & v_wall_ = (cartesian velocities v_x_ & v_y_ & v_z_ = N).

It is worth noting that the conditions BC1 and BC2 imply that the pressure may be developed in the screw/die system. However, since the pressure at the screw element end is unknown (at the die exit it is equal to zero), the pressure gradient may be calculated in this case only.

The Ostwald-de Waele model was used for process modeling with Polyflow:

(2) $$\eta =m{\dot{\gamma }}^{n-1}$$

where *η* is the viscosity, Pa·s, $\dot{\gamma }$ is the shear rate, s^−1^, *m* is the coefficient of consistency, Pa·s^n^, *n* is the flow exponent.

The coefficient of consistency was equal to *m* = 30,100 Pa·s^n^, and the flow exponent was equal to *n* = 0.25.

The Navier law [51] was used for slip modeling with Polyflow:

(3) $$\tau_{w}=F_{slip}\left(U_{wall}-U_{s}\right){\left|U_{s}-U_{wall}\right|}^{e_{slip}-1}$$

where *τ_w_* is the shear stress, *U_s_* is the fluid velocity in the tangential direction, *U_wall_* is the wall velocity in the tangential direction (*U_wall_* = 0 by default), and *F_slip_* and *e_slip_* are the model parameters (the full slip appears when *F_slip_* = 0, the model is linear when *e_slip_* = 1).

The parameters of the Navier equation were obtained using rheometric data presented in Figure 3, which were approximated using a power-law model:

(4) $$\tau_{w}=F_{slip}U_{s}{}^{e_{slip}}$$

where *τ_w_* is the shear stress, N/mm^2^, *U_s_* is the slip velocity, mm/s, and *F_slip_* = 0.064 and *e_slip_* = 0.43 are the model parameters.

**Figure 6:**
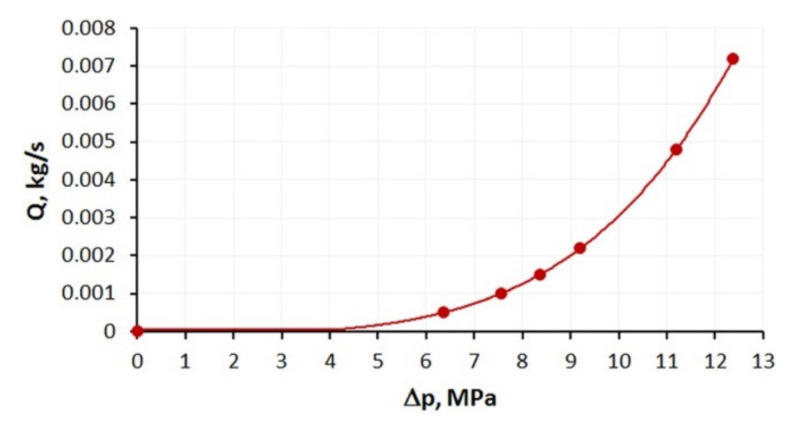
Die characteristics (*F_slip_* = 0.064, *e_slip_* = 0.43).

**Figure 7:**
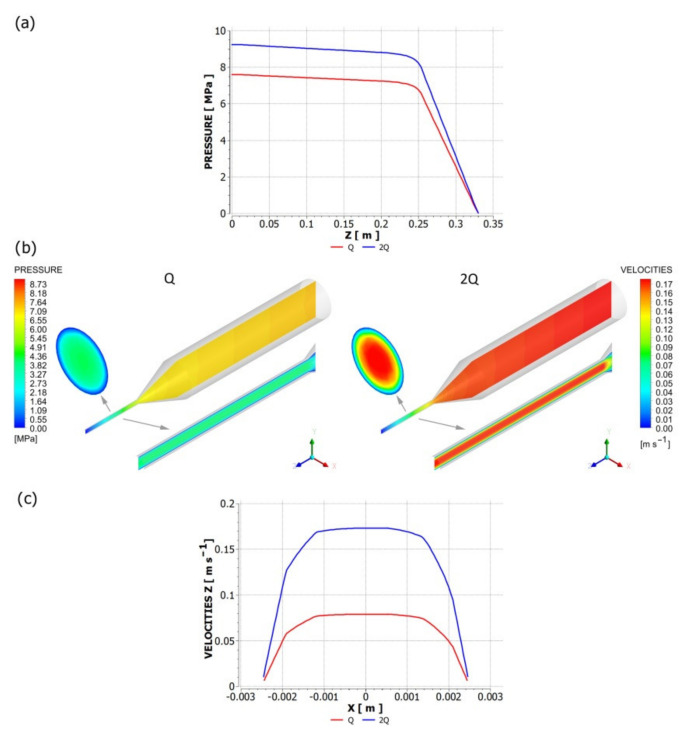
Pressure and velocity distributions for die flow (*F_slip_* = 0.064, *e_slip_* = 0.43): *Q* = 0.001 kg/s. (**a**) pressure profile, (**b**) velocity distribution (in cross-sections indicated by arrows), and pressure distribution, (**c**) velocity profile.

An influence of slip parameters (*F_slip_* = 0.064, *e_slip_* = 0.43) on the polymer flow in the die is presented in Figure 6 where die characteristics are shown. The cylindrical, three-zone die of diameter D = 45 mm at the onset and diameter D = 5 at the end was used in the study. The lengths of die sections were equal to L = 180 mm. 30 mm, 60 mm (Figure 5a). Various flow rates were used for simulations: Q = 0.0005 kg/s, 0.001 kg/s, 0.0015 kg/s, 0.0021 kg/s, 0.0048 kg/s, 0.0072 kg/s. A non-linear dependence of the flow rate on the die pressure drop is clearly seen. An example of pressure and velocity distributions is shown in Figure 7. Slipping at the die surface is very low.

**Figure 8:**
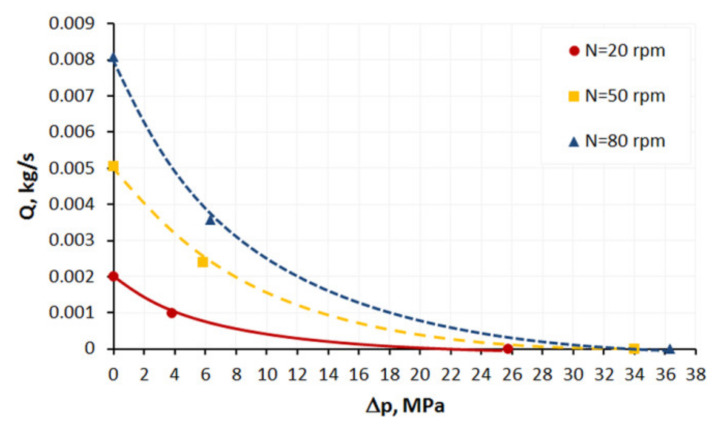
Screw characteristics at varoius screw speed (*F_slip_* = 0.064, *e_slip_* = 0.43).

**Figure 9:**
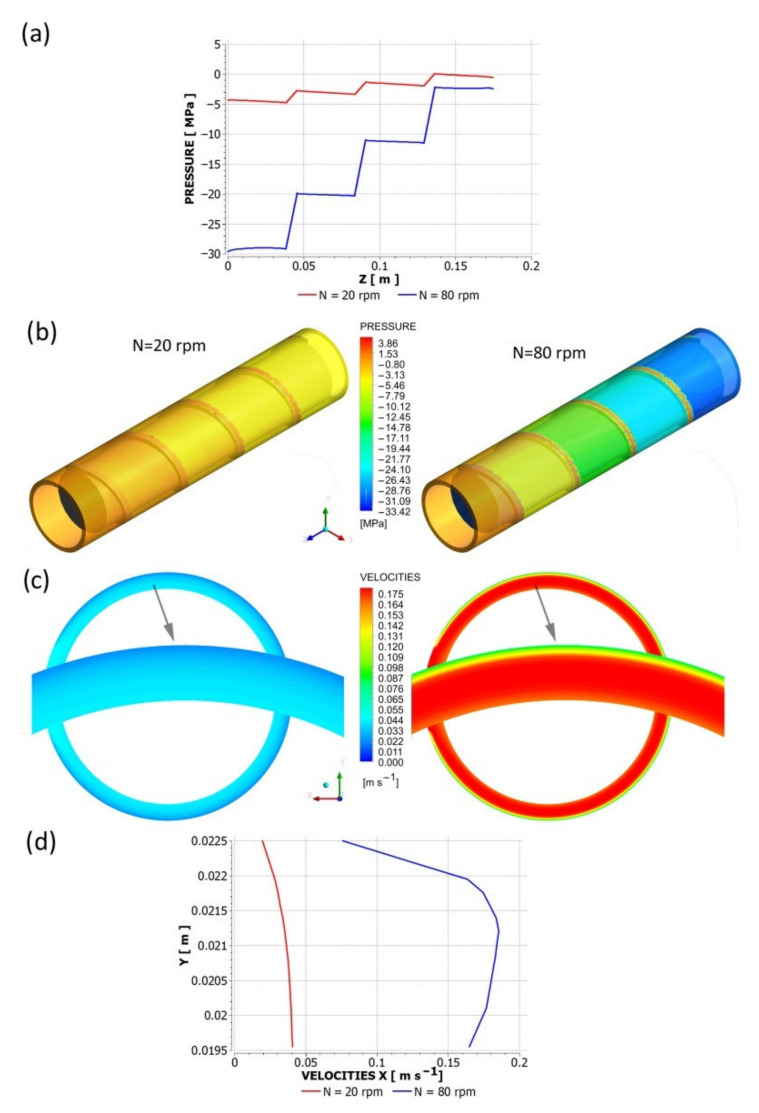
Pressure and velocity distributions for screw flow (*F_slip_* = 0.064, *e_slip_* = 0.43): Q = 0.001 kg/s. (**a**) pressure profile, (**b**) pressure distribution, (**c**) velocity distribution, (**d**) velocity profile.

An influence of slip parameters (*F_slip_* = 0.064, *e_slip_* = 0.43) on the polymer flow in the screw is presented in Figure 8 where screw characteristics are shown. The conventional screw geometry was used in the study with the screw element of diameter *D* = 45 mm, the depth of the screw channel *H* = 3 mm, and the length *L* = 180 mm (Figure 5b). Various flow rates were used for simulations: *Q* = 0.001 kg/s, 0.0021 kg/s, 0.0048 kg/s, 0.0072 kg/s. A non-linear dependence of the flow rate on the screw pressure increase is clearly seen. When screw speed increases, the flow rate increases at the constant pressure, and the pressure increases at the constant flow rate. The drag flow can be evaluated as the flow rate at the pressure change Δ*p* = 0. An example of pressure and velocity distributions is shown in Figure 9. Slipping is clearly seen at the screw and barrel surfaces.

## 4. Extrusion Modeling

Extrusion is the system in which the extruder co-operates with the die. The flow in the extruder affects the flow in the die, and the flow in the die influences the flow in the extruder.

Any changes in the flow conditions in the extruder cause changes in the flow conditions in the die, and any changes in the flow conditions in the die cause changes in the flow conditions in the extruder. The global extrusion process modeling requires taking into account this extruder/die co-operation.

The global model of the extrusion process is composed of the elementary models which describe the flow in the screw, that is in the solid conveying zone, in the delay zone, in the melting zone, and finally in the metering zone, as well as in the die zone. The calculations are carried out in the screw increments, where the parameters of the process, e.g., temperature or pressure, at the exit of the current increment are the inlet data for the next increment.

The extrusion global models use various procedures of computation which should be suitable for the modeled process, which is discussed, e.g., in [30,52,53]. In the extrusion with polymer flood feeding, the forward procedure of calculations is used. The extrusion throughput is not known here, and it is calculated by iteration searching for the extruder operating point which defines the extrusion throughput and the die pressure (the extrusion pressure). In the extrusion with polymer metered feeding, the inverse backward procedure of calculations is used, the flow rate is set here and equals the flow rate in the dosing device. The issue of global modeling was recently discussed in details by the authors [36].

As it was said, the global model of the extrusion process is composed of elementary models for the screw and the model for the die. The polymer mass flow rate is constant in the screw/die increments, and the process parameters may be assumed locally constant in these increments. This modeling approach is called the lumped parameter modeling which is accurate enough for engineering practice and applications.

The parameters of extrusion process, e.g., flow rate, temperature, power, pressure, screw filling, at the onset of the current screw increment are equal to the parameters at the exit of the former increment, i.e.,

(5) f_i−1_out_ (*z*) = f_i_in_ (*z*)

(6) f_i−1_out_ (*z* + Δ*z*) = f_i_in_ (*z* + Δ*z*)

where f_i_in_ (*z*)—input data (temperature, pressure, or solid material content) at the onset of *i*—increment, f_i−1_out_ (*z*)—output data at the exit of (*i* − 1)—increment, *z*—coordinate along the screw/die axis, Δ*z*—screw/die increment.

The extrusion model that is discussed here is an extended version of the previous models [4], with novel concepts for slip modeling using three-dimensional, non-newtonian flow characteristics for the screw/die system.

Summarizing the model presented here is composed of the elementary models:

- Solid conveying section;
- delay section;
- melting section, the model is dependent on the filler content, a one-dimensional model for less than 50% filler is used, a two-dimensional model for over 50% filler is used (Figure 1);
- screw melt conveying section including slip effects using three-dimensional, non-Newtonian flow characteristics;
- die flow section with slip effects using three-dimensional, non-Newtonian flow characteristics.

**Figure 10:**
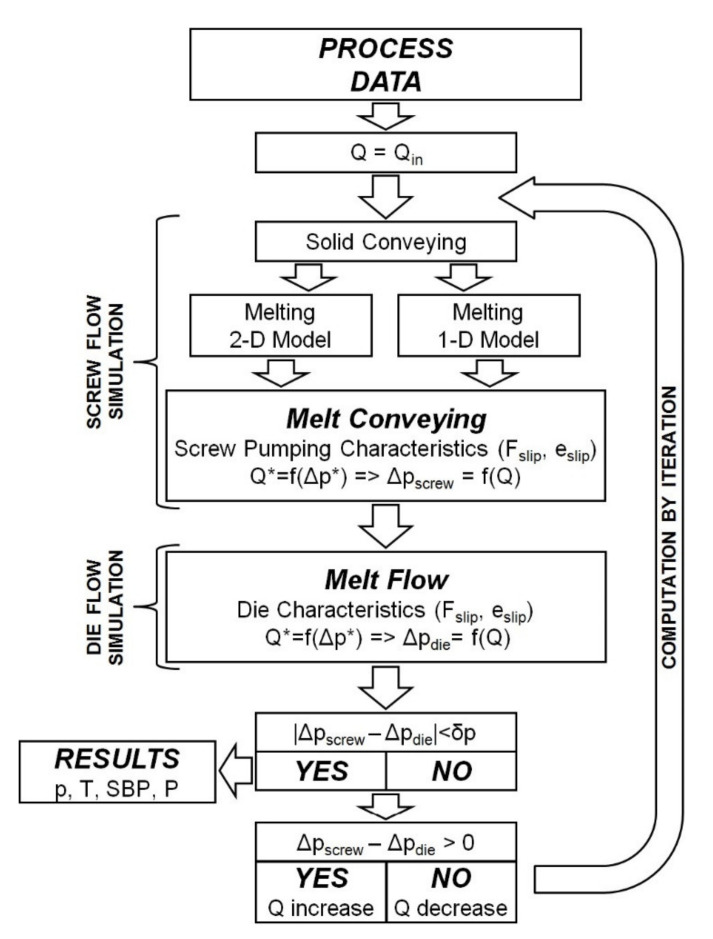
Algorithm of calculation: Q—polymer flow rate (extrusion throughput), Q_in_—initial polymer flow rate, Δp_screw_—pressure build-up in the screw, Δp_die_—pressure drop in the die, p—pressure, T—temperature, SBP—solid bed profile, P—power.

The calculation procedure is depicted in Figure 10. The conventional process with polymer flood feeding is considered here, and thus the forward scheme of calculation is applied. The computations begin with an initial mass flow rate. Then the process is modeled using different melting models (one-dimensional or two-dimensional). Afterwards, the flow characteristics with slip effects are used for flow modeling in the screw/die system. The exit screw pressure is checked for the convergence with the die pressure. Then, the polymer flow rate is changed (increased or decreased) according to the result of comparison, which can be positive or negative. The calculations are reiterated until the convergence of calculation has been reached.

The screw pumping characteristics were discussed in detail in [20]. In this paper, these characteristics have been approximated by the formula

(7) $$Q^{*}=a-bn^{c}\Delta p^{*}$$

where Q* is the flow rate in the dimensionless form, Δp* is the pressure in the dimensionless form, and *a*, *b*, *c* are the model parameters (*a* = 1.003, *b* = 3.195, *c* = 0.561).

## 5. Process Simulations

A classical screw with diameter D = 45 mm and length to diameter ratio (L/D) = 27 was applied. The lengths of the screw zones were equal to L_F_ = 485 mm (the feeding zone), L_C_ = 320 mm (the compression zone), L_M_ = 410 mm (the metering zone), and the total screw length was equal to L = 1215 mm. The depths of the screw channel in the zones were equal to H_F_ = 8 mm (the feeding zone), H_M_ = 3 mm (the metering zone), and the compression ratio which is defined as the ratio of the screw channel depth in the feeding zone to the screw channel depth in the metering zone was equal to CR = H_F_/H_M_ = 2.66. The pitch of the screw was equal to the screw diameter, i.e., t = D, so the helix angle φ was equal to 17.65°. The width of the screw flight was equal to e = 5 mm. The die for cylindrical rods of diameter D = 5 mm was applied in the study. Simulations were performed for various screw speeds N = 20 rpm, 50 rpm, and 80 rpm.

The rheological equation of Klein was used for the viscosity description.

(8) $$\ln\eta =a_{0}+a_{1}\ln\dot{\gamma }+a_{11}\ln^{2}\dot{\gamma }+a_{12}\ln\dot{\gamma }T+a_{2}T+a_{22}T^{2}$$

where: *η* is the viscosity, Pa·s, $\dot{\gamma }$ is the shear rate, s^−1^, T is the temperature, °C, and *a*_0_, *a*_1_, *a*_11_, *a*_12_, *a*_2_, *a*_22_ are the model parameters (*a*_0_ = 40.12992893, *a*_1_ = −0.85791717, *a*_11_ = −0.01421715, *a*_12_ = 0.00160931, *a*_2_ = −0.27500818, *a*_22_ = −0.00060042).

**Figure 11:**
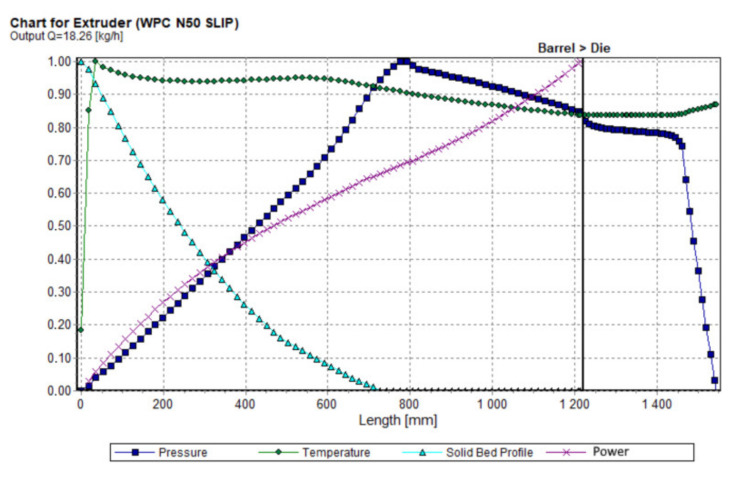
Overall extrusion process characteristics.

**Figure 12:**
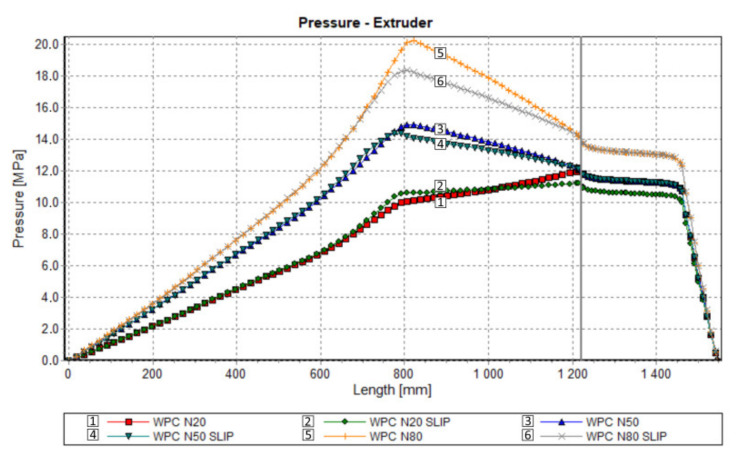
Process simulation: Pressure profile.

**Figure 13:**
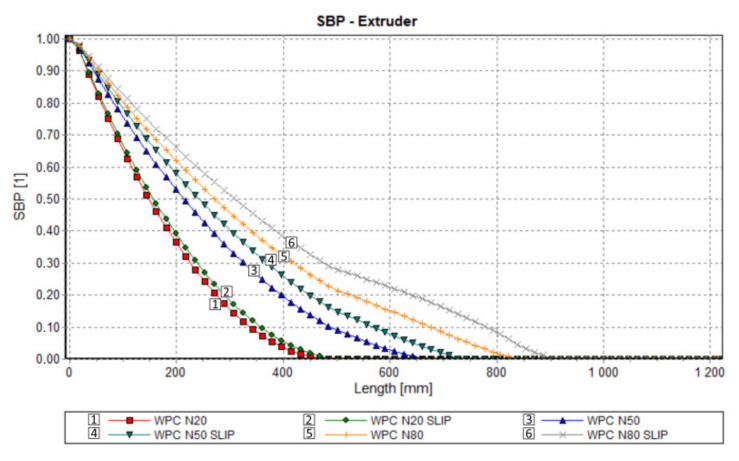
Process simulation: Melting profile.

**Figure 14:**
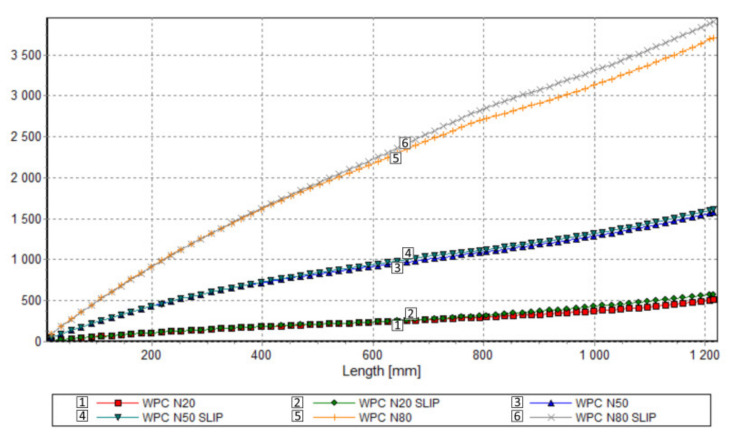
Process simulation: Power consumption profile.

Results of computation are shown in Figure 11, Figure 12, Figure 13 and Figure 14. The overall extrusion process characteristics are depicted in Figure 11. These dimensionless characteristics are composed of the important process parameters, i.e., the pressure, the temperature, the solid bed content, and the power.

An effect of slip on the process flow is clearly seen in Table 1 and Figure 12, Figure 13 and Figure 14. Table 1 shows that the flow rate (extrusion throughput) increases when slip appears. The pressure decreases when slip appears (Figure 12), and melting is slower in this case, which may be caused by an increase in the flow rate (Figure 13). Power consumption is a bit higher in the case when slip appears, which may also result from an increase in the flow rate (Figure 14).

## 6. Experimental

Validation of simulations has been performed in this paper at the process simulation data, which were presented above. Various screw speeds N = 20 rpm, 50 rpm, and 80 rpm were applied, and the barrel/die temperature profile was set as T = 185 °C, 190 °C, 190 °C, 170 °C. The pressure was measured along the screw, and in the die.

**Figure 15:**
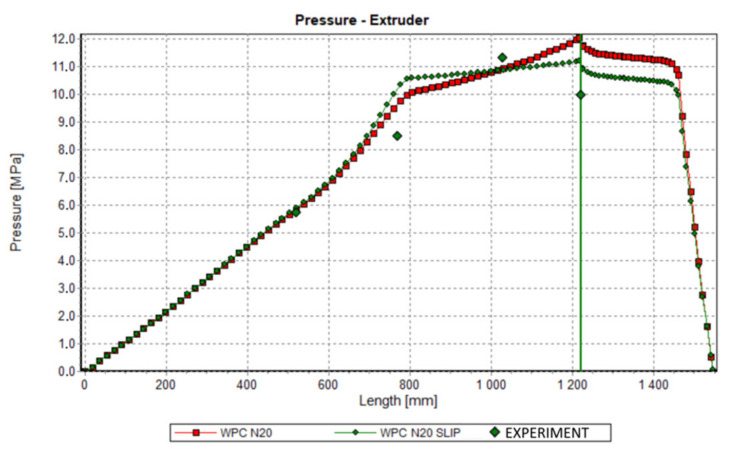
Validation of simulation: Pressure profile, N = 20 rpm.

**Figure 16:**
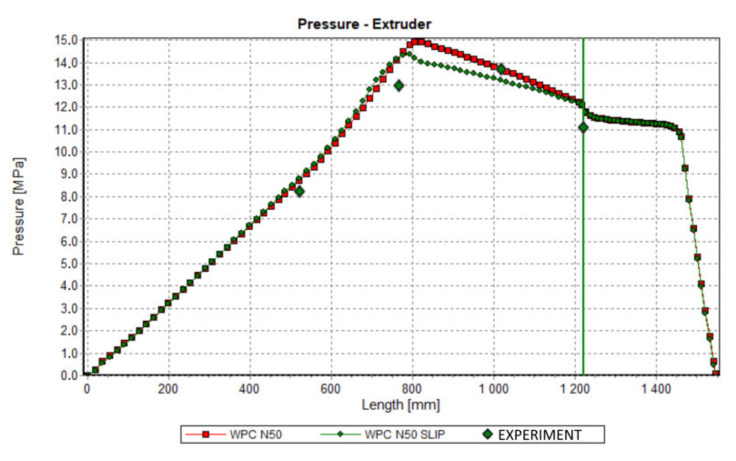
Validation of simulation: Pressure profile, N = 50 rpm.

**Figure 17:**
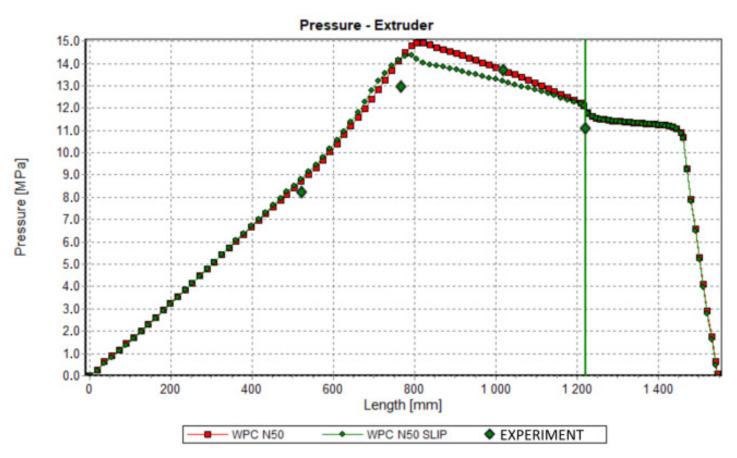
Validation of simulation: Pressure profile, N = 80 rpm.

The results of validation are presented in Figure 15, Figure 16 and Figure 17. The predictions were quite good, although the simulations were generally overestimated comparing to experiment, and some discrepancies in a few experimental points were observed between the simulation and experiment. For higher screw speeds, N = 50 rpm and N = 80 rpm, the influence of including the slip effects into the model is clearly seen. The computed pressure decreases, and the pressure profile shifts towards the experimental data. For the lower screw speed, N = 20 rpm, the slip effect on modeling is not clear. Two reasons for this may be considered, that is the effect of yield stress and the elongational viscosity (important in the conical die), which may occur at the low shear/elongation rates, and which is not included in the model.

## 7. Conclusions

So far, any global model of extrusion of wood polymer composites that would include slip effects was not known. In this paper, slip effects were considered and discussed both for WPC rheological studies as well as for WPC extrusion modeling.

The concept of the global model of single screw extrusion process of wood polymer composites has been discussed with respect to slip effects, extensively. Two aspects of slip effects have been considered, rheological and extrusion flow modeling. In the first case, the effect of slipping has to be removed from viscosity measurements. This can be performed by using the Mooney correction. In the second case, slipping in the extrusion process has to be considered. Both these effects can be called the rheological slip effect and the extrusion process slip effect.

Removing the slip effects from viscosity measurements results in increasing the viscosity since the shear rate decreases with the shear stress unchanged. This may have substantial effects in process modeling. From the other side, considering the slip effects in process modeling results in increasing the flow rate and pressure decrease. Both these effects should be considered when modeling the global extrusion process, and the final result is dependent on the current material and process parameters.

Modeling of the single screw extrusion process of wood polymer composites with slip effects is not a trivial problem. It is difficult to determine the slip parameters using the capillary rheometers, and then to model and simulate the extrusion process.

The rheological characteristics of wood polymer composites are generally not available in the material data bases, like CAMPUS or Autodesk MOLDFLOW. The (WPC) material characteristics are strongly dependent on the material structure, and the size of the fiber or flour. The smaller the size of the filler, the easier and more reliable the measurement. Proper preparation of the material is very important. The results are generally difficult to reproduce. When the material is more homogeneous, the results are more reproducible. The temperature flow range is narrow, and it is difficult to determine the melting point. Therefore, the concept of no flow temperature is used to determine the onset of the flow.

The concept of modeling of extrusion of wood polymer composites presented here requires validation in a broader range of material and process parameters, both in relation to the rheological characteristics of the material and the processing conditions. The research will be continued in this direction.

Finally, it is important to note that the concepts of flow modeling with slip effects presented in this paper, i.e., rheological/flow modeling issues, may be applied or extended to other material processing systems, e.g.,

- Processing of the materials characterized by wall slip,
- processing of filled/reinforced polymeric materials (by extrusion and injection molding),
- food and cosmetics processing,
- pharmaceutical industry [54],
- 3D printing [55,56,57].

## Figures and Tables

**Table 1:** Research program.

**Screw Speed, rpm**	**Throughput (Predicted), kg/h**	
	No Slip	Slip
20	07.94	08.41
50	16.05	18.26
80	27.87	31.69

## Data Availability

The data presented in this study are available on request from the corresponding author.
